# Inhibition of the mechano-enzymatic amyloidogenesis of transthyretin: role of ligand affinity, binding cooperativity and occupancy of the inner channel

**DOI:** 10.1038/s41598-017-00338-x

**Published:** 2017-03-15

**Authors:** Guglielmo Verona, P. Patrizia Mangione, Sara Raimondi, Sofia Giorgetti, Giulia Faravelli, Riccardo Porcari, Alessandra Corazza, Julian D. Gillmore, Philip N. Hawkins, Mark B. Pepys, Graham W. Taylor, Vittorio Bellotti

**Affiliations:** 1 Wolfson Drug Discovery Unit, Centre for Amyloidosis and Acute Phase Proteins, University College London, London, NW3 2PF UK; 2 Department of Molecular Medicine, Institute of Biochemistry, University of Pavia, Via Taramelli 3b, Pavia, 27100 Italy; 3 Department of Medical and Biological Sciences (DSMB), University of Udine, Piazzale Kolbe 4, 33100 Udine, Italy; 4 National Amyloidosis Centre, University College London, London, NW3 2PF UK

## Abstract

Dissociation of the native transthyretin (TTR) tetramer is widely accepted as the critical step in TTR amyloid fibrillogenesis. It is modelled by exposure of the protein to non-physiological low pH *in vitro* and is inhibited by small molecule compounds, such as the drug tafamidis. We have recently identified a new mechano-enzymatic pathway of TTR fibrillogenesis *in vitro*, catalysed by selective proteolytic cleavage, which produces a high yield of genuine amyloid fibrils. This pathway is efficiently inhibited only by ligands that occupy both binding sites in TTR. Tolcapone, which is bound with similar high affinity in both TTR binding sites without the usual negative cooperativity, is therefore of interest. Here we show that TTR fibrillogenesis by the mechano-enzymatic pathway is indeed more potently inhibited by tolcapone than by tafamidis but neither, even in large molar excess, completely prevents amyloid fibril formation. In contrast, mds84, the prototype of our previously reported bivalent ligand TTR ‘superstabiliser’ family, is notably more potent than the monovalent ligands and we show here that this apparently reflects the critical additional interactions of its linker within the TTR central channel. Our findings have major implications for therapeutic approaches in TTR amyloidosis.

## Introduction

The seminal observation that the native non-covalent TTR homotetramer dissociates at low pH into dimers and monomers that self-assemble into amyloid fibrils^1^ is the basis for the current, widely accepted model for TTR amyloid formation. However low pH treatment of both wild type and amyloidogenic TTR variants produces mostly heterogeneous amorphous aggregates with a very low yield of authentic amyloid fibrils showing pathognomonic green birefringence in polarized light after Congo red staining, characteristic fibrillar electron microscopic appearance and the cross-β X-ray fibre diffraction signature. *Ex vivo* TTR amyloid deposits, especially in the heart, usually contain a substantial proportion of the *C*-terminal TTR fragment generated by proteolytic cleavage at Lys48-Thr49 ^2^. Following this observation we have identified and characterized a novel mechanism of TTR amyloid fibrillogenesis mediated by selective tryptic cleavage at residue 48. The highly amyloidogenic TTR *C*-terminal residue 49–127 polypeptide is released, catalysing amyloid fibril formation *in vitro*, and the whole process is strongly enhanced by biomechanical forces^3, 4^. Abundant authentic amyloid fibrils are produced with pathognomonic features indistinguishable from natural *ex vivo* fibrils.

Compounds able to stabilize TTR, intended for use as drugs to treat and prevent systemic ATTR amyloidosis, have hitherto been identified exclusively by their capacity to inhibit TTR dissociation and aggregation induced by low pH *in vitro*. However, we have found that inhibition of the mechano-enzymatic pathway of TTR amyloid fibrillogenesis at physiological pH, ionic strength and temperature requires occupation of both thyroxine binding sites in each native TTR tetramer. This is most efficiently achieved by bivalent ligands, exemplified by the palindromic compound mds84^5^, that is spontaneously bound simultaneously and pseudo-irreversibly in both sites. Sant’Anna and colleagues^6^ have recently reported that tolcapone is a potent inhibitor of TTR dissociation and aggregation under denaturing conditions. They attribute its efficacy to occupation of both binding pockets with similar high affinity in contrast to the notable negative cooperativity observed with thyroxine itself and other monovalent ligands, including the TTR stabiliser drugs, tafamidis^7, 8^ and diflunisal^9^, which are now in clinical use. This important observation prompted us to test the capacity of tolcapone to inhibit mechano-enzymatic amyloidogenesis of TTR using the V122I variant which is the most prevalent cause of hereditary cardiac amyloidosis^10^.

## Results and Discussion

**Figure 1:**
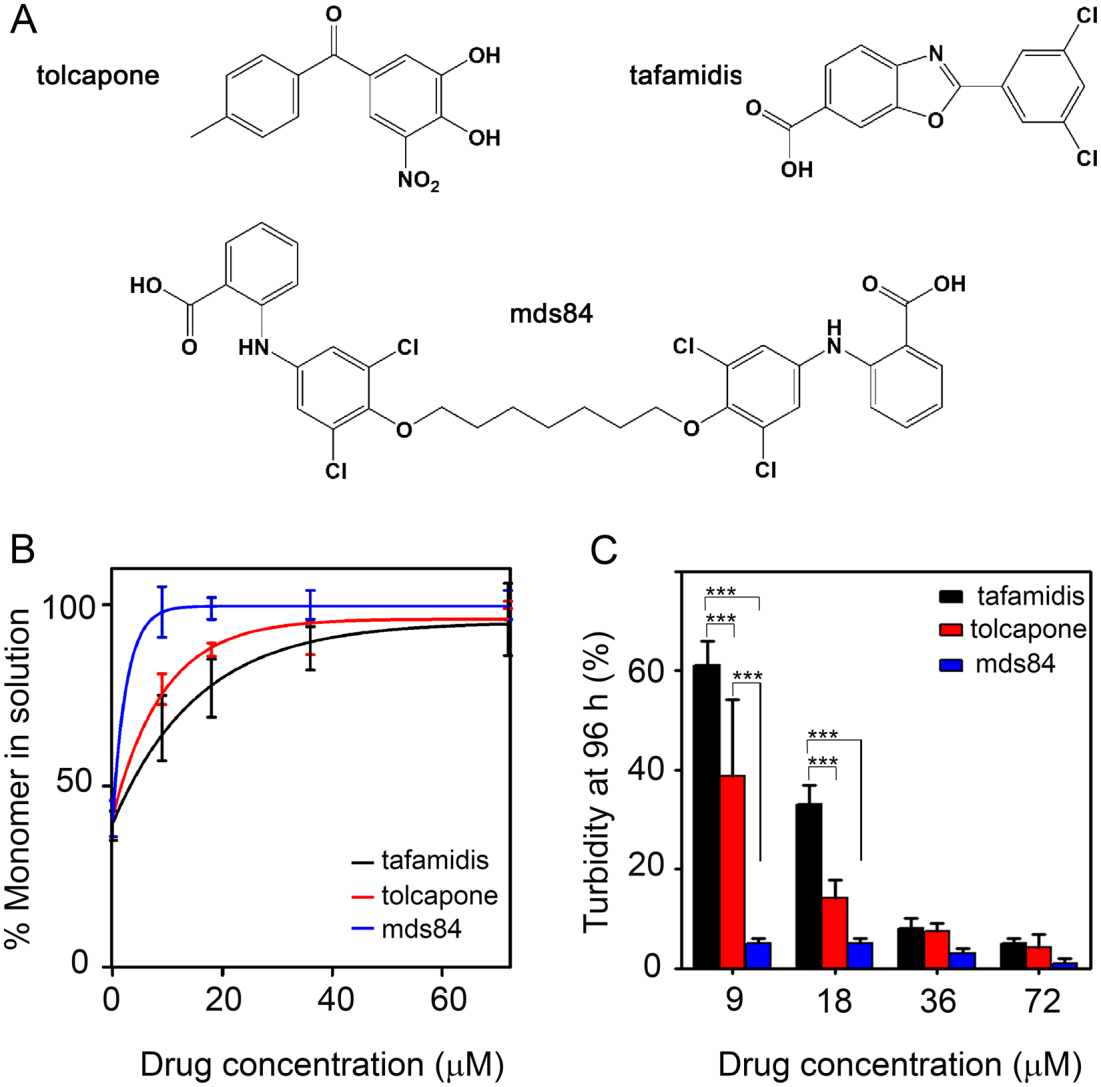
Comparative effect of tolcapone on proteolysis and fibrillogenesis of V122I TTR. (**A**) Chemical structures of tolcapone and tafamidis together with the TTR binding palindromic ligand, mds84. (**B**) Aggregation of 18 µM V122I TTR in the presence of 0, 9, 18, 36 and 72 µM of tolcapone, tafamidis and mds84 respectively in PBS pH 7.4 at 37 °C with fluid agitation was carried out after addition of trypsin at an enzyme:substrate ratio of 1:200. Selective proteolytic cleavage was monitored at 96 h by SDS-PAGE under reducing conditions (Supplementary Fig. S1). Intensities of the electrophoretic bands corresponding to the intact protomer in the whole mixture were normalized to 100% for the same band of the protein before addition of trypsin. The solid lines represent the nonlinear fit to the experimental mean (SD) of three replicates using GraphPad Prism v5. Two way ANOVA gave a P value < 0.001 for tafamidis vs mds84 at 9 and 18 µM; for tolcapone vs mds84 at 9 µM. (**C**) Aggregation of 18 µM V122I TTR was quantified as spectrophotometric turbidity at 400 nm normalized to 100% for aggregation of the protein in the absence of ligands. We know from previous work that TTR aggregation in this system is in the form of authentic amyloid fibrils^3, 4^. All data shown represent mean (SD) of three independent experiments, and *** represents P < 0.001.

We compared tolcapone with tafamidis^7, 8^ and the experimental bifunctional compound, mds84^5^ (Fig. 1A). When stirred with V122I TTR at 37 °C in PBS in the presence and absence of trypsin for 96 h, all three ligands inhibited proteolytic cleavage of the protein in a dose-dependent manner (Fig. 1B and Supplementary Fig. 1). At molar ratios of ligand:TTR tetramer higher than 2:1, the three compounds had similar efficacy with apparent almost complete inhibition of TTR cleavage. However at lower molar ratios, mds84 was the most potent inhibitor, followed by tolcapone and tafamidis.

Consistent with their inhibition of proteolytic TTR cleavage, the three compounds also inhibited fibril formation (Fig. 1C). At a ligand:TTR tetramer molar ratio of 0.5:1, mds84 and tolcapone reduced fibril formation by approximately 90% and 60% respectively, and tafamidis only by 40%. At molar equivalence, inhibition increased at 60% with tafamidis, ~80% with tolcapone and remained at ~90% with mds84. At twofold and greater molar excess of ligand, both monovalent ligands, tafamidis and tolcapone inhibited TTR fibrillogenesis by the same amount (~90%) as mds84.

Even when we compared the effect of the three compounds on the inhibition of TTR acidic-mediated aggregation^11^, mds84 at equimolar concentration with TTR was the only ligand able to completely inhibit the process (Supplementary Information and Supplementary Fig. S2).

The inhibition of the mechano-enzymatic pathway of TTR amyloidogenesis, which we believe to be the most likely pathophysiological mechanism *in vivo*, depends on the occupation of both thyroxine binding sites. The superior potency of tolcapone among monovalent TTR ligands is consistent with its unique property of not inducing negative cooperativity^6^.

The much more effective inhibition of proteolysis-mediated TTR fibrillogenesis by mds84 results from the simultaneous occupation of both thyroxine binding sites, and the internal channel between them, by this palindromic molecule^5^. It occurs rapidly and completely at equimolar concentrations of TTR and ligand. Binding of mds84 by native TTR is pseudo-irreversible under physiological conditions^5^ and generates a stable complex. This in contrast to the reversible monovalent ligands for which a higher molar excess is required to saturate both binding sites *in vivo*. These limitations on efficacy against mechano-enzymatic mediated amyloidogenesis may explain the modest therapeutic benefit of monovalent ligands in the clinical studies reported so far^12^.

**Figure 2:**
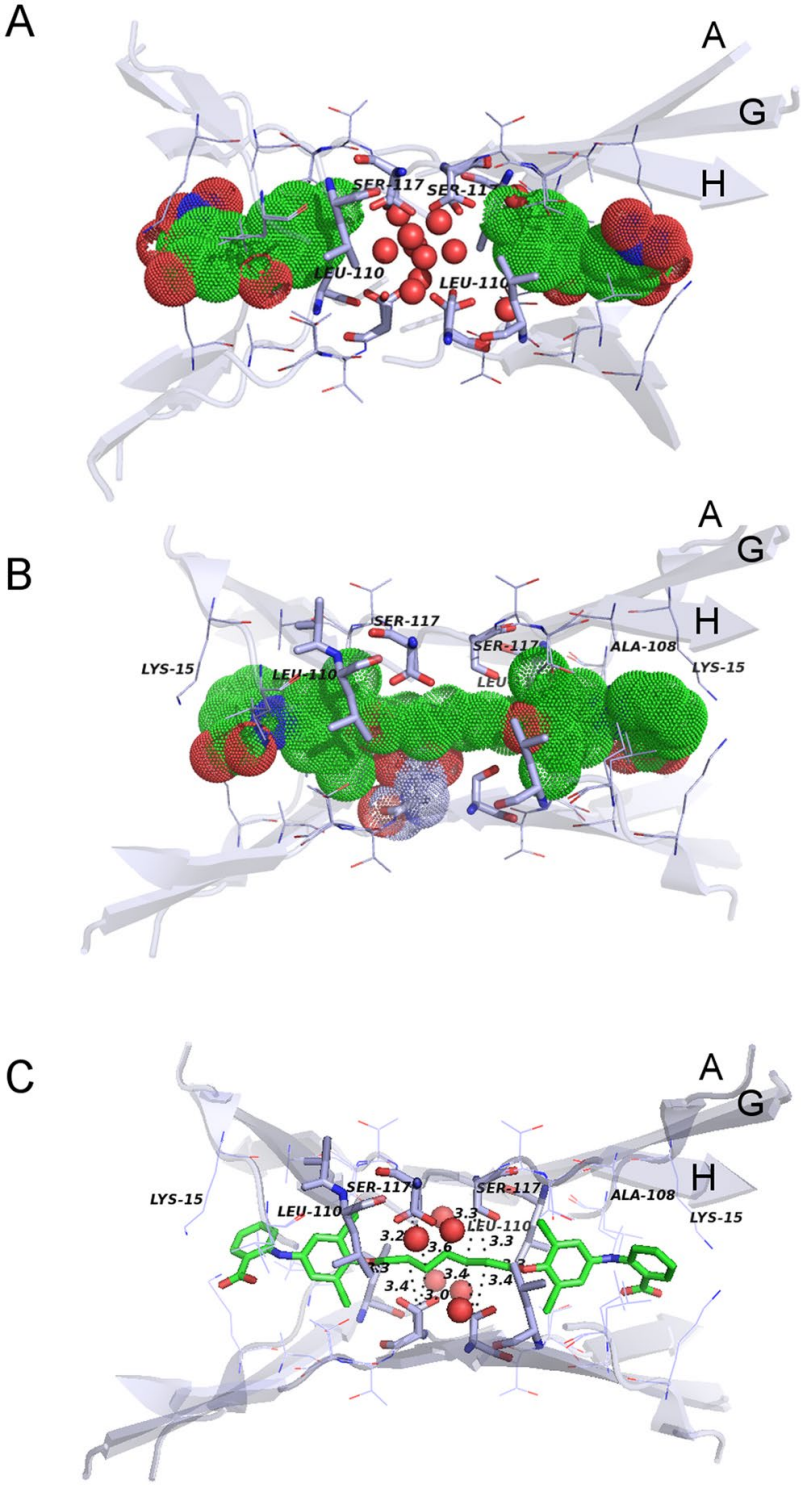
TTR binding sites in the presence of tolcapone and mds84. Wild type TTR binding sites occupied by tolcapone (**A**) and mds84 (**B**) with ligands shown as solvent accessible surfaces. For clarity, H_2_O oxygens are shown as spheres with 50% of the van der Waals radius. (**C**) Wild type TTR-mds84 complex with highlighted distances of the principal hydrophobic contacts between the ligand methylene linker and TTR atoms.

To understand the superior inhibitory effect of bivalent ligands, we analysed the deposited X-ray structures of TTR complexed with monovalent ligands, tolcapone^6^ and tafamidis^7^, (Fig. 2) in comparison with bivalent ligands, mds84^5^ and compounds 20 and 22 of Green *et al.* ^13^ (Supplementary Fig. S3). For a general description of the crystal structures we refer to a very exhaustive review by Palaninathan^14^. There is no PDB structure of the mds84-V122I TTR complex so the analysis is restricted to wild type TTR. This choice is justified by the almost perfect superposition of the wild type and V122I TTR structures in the absence of ligands (Supplementary Fig. S4A,B) with minor deviations when bound to tolcapone (Supplementary Fig. S4C,D). The average root mean square deviations (rmsd) for the TTR backbone residues are 0.45 and 0.9 Å for the two proteins in their free and bound form, respectively. Moreover, Val122 and Ile122 side chains point towards the external part of the pocket cavity and residue 122 is not present in the halogen binding pockets (HBPs; see Methods). Importantly, neither mono- nor bivalent molecules induce major rearrangements of the HBP residues with rmsd values calculated for the backbone atoms of HBP1, HBP2 and HBP3 ranging from 0.2 to 0.3 Å (Table 1). The superposition of the binding pockets of TTR with and without ligands is shown in Supplementary Fig. S5. Also there is no correlation between the number of ligand-protein hydrogen bonds, (Table 1) and inhibitory efficacy. A full list of hydrogen bonds between the ligand and the HBPs is reported in the Supplementary Information. However, in contrast with the monovalent ligands which interact only with the ligand pocket itself, the methylene linker of the bivalent compounds occupies the narrow TTR inner channel with an interaction surface increased by 48 Å^2^, for mds84 compared with tolcapone. Binding of mds84^5^, compound 20 and compound 22^13^ by TTR creates, respectively, up to 9, 5 and 4 hydrophobic interactions, mainly with S117 Cβ and Leu110 Cδ2. These interactions make an estimated favourable energetic contribution of −4.6, −2.5 and −1.0 kcal/mol for the three ligands respectively. Occupancy of the central channel of TTR, estimated for mds84 to be around 80% of the void volume, may thus have a crucial role in preventing subunit sliding and also provide long distance stabilisation affecting the dynamics of CD loop, the selective cleavage of which potently primes amyloid fibrillogenesis. The pioneering study of Green *et al.* ^13^ identified the enhanced stabilisation of TTR by bivalent ligands with a central linker but their compounds did not interact with native TTR. They were bound only by renaturation of dissociated denatured TTR around the ligand.^13^ In sharp contrast, both the mds84 prototype and its analogues are rapidly bound by the native TTR tetramer, traversing the inner channel to occupy it with the linker.^5^ The unique total binding site in TTR, comprising the two halogen binding pockets and the inner channel, is entered by these ligands with a decreased entropic cost compared to all monovalent ligands^15^. The key factors responsible for the ‘superstabiliser’ property of mds84 and its analogues are thus likely to be the combination of entropic gain, increased hydrophobic contributions of the linker and a reduced void volume in the interface cavity.

**Table 1:** Structural comparisons of TTR halogen binding pockets with and without ligands.

Protein (PDB code)	*Rmsd (Å)	**Numbers of H bonds
WT TTR/tolcapone (4d7b)	0.247	3
WT TTR/tafamidis (3tct)	0.196	0
WT TTR/mds84 (3ipe)	0.173	2
WT TTR/compound 20 (2fbr)	0.298	6
WT TTR/compound 22 (2flm)	0.319	0

*The root mean square deviation was calculated for the backbone atoms of all the three HBPs for the wild type TTR complexes compared to the same protein without ligand (PDB 1dvq). **The hydrogen bonds considered are those between the ligand and the HBPs atoms including crystallographic water molecules.

The mechanism of binding of TTR by bivalent compounds cannot be fully clarified on the basis of the available crystallographic structures. These data reveal that the TTR inner cavity is very narrow and therefore apparently inaccessible to the bulky groups of the ligands. We hypothesize that the tetramer dynamics may be larger than expected and current studies in our laboratory using NMR might shed light on this important issue.

**Figure 3:**
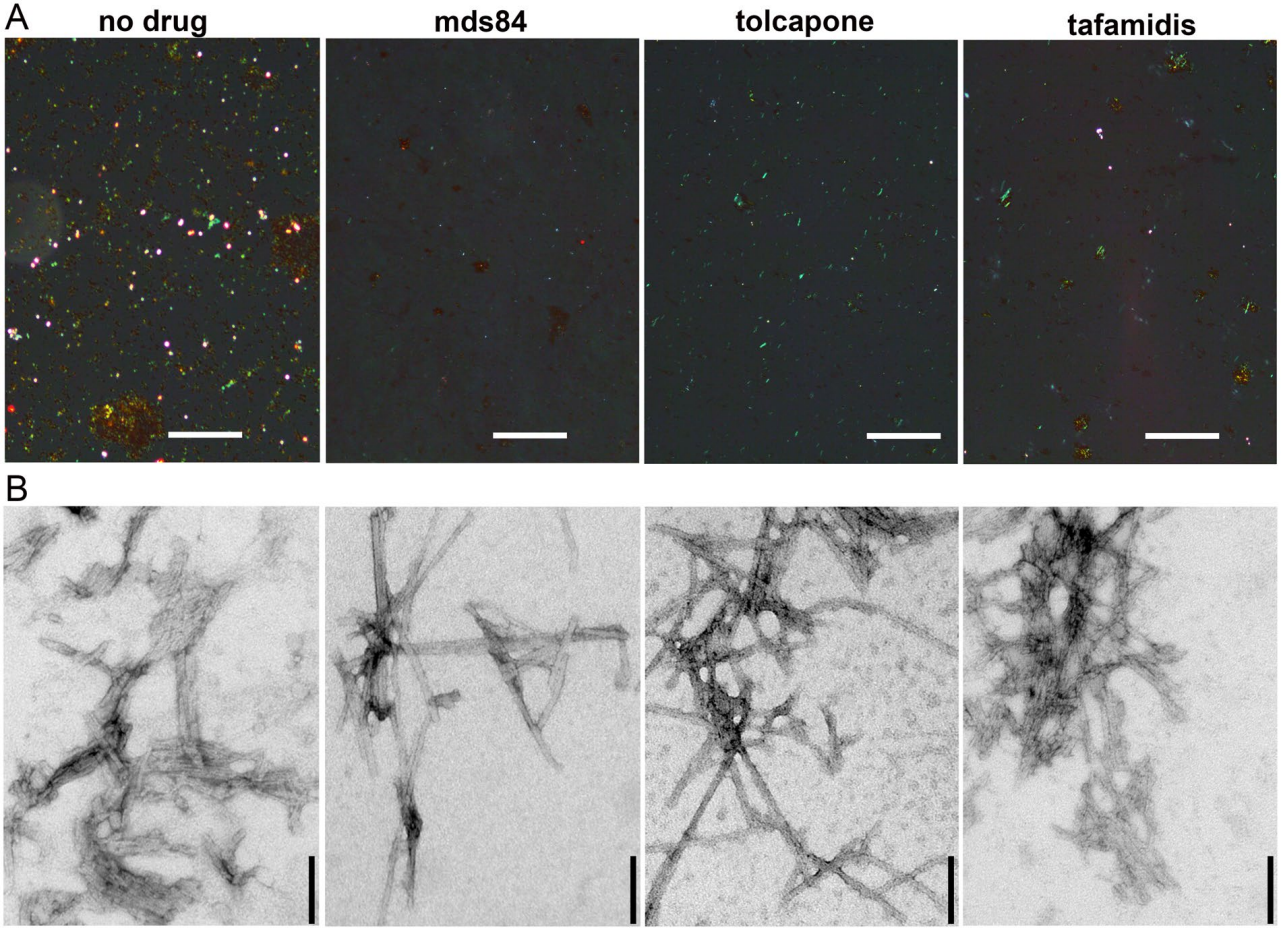
Residual amyloid aggregates in the presence of excess of ligands. (**A**) Congo-red stained specimens viewed under intense cross polarized light in the absence of any ligands and in the presence of fourfold molar excess of mds84, tolcapone and tafamidis (Supplementary Fig. S6). Some fragments of amyloid are present with maximally inhibitory ligand concentrations (Fig. 1), although least with mds84. Scale bar, 100 µM. (**B**) Typical fibrillar structures detected by exhaustive analysis of negatively stained electron microscopy images of the same TTR-ligand preparations. Scale bar, 100 nm.

Finally, even at large molar excess of tafamidis or tolcapone that inhibited obvious *in vitro* aggregation, ultracentrifugation of the reaction mixture yielded a small amount of insoluble material which stained with Congo red to give typical green birefringence (Fig. 3A and Supplementary Fig. S6) and was fibrillar in the electron microscope (Fig. 3B). In the presence of mds84 such insoluble material was minimal but extensive searching by electron microscopy revealed some fibrils (Fig. 3B). Evidently, the mechano-enzymatic mechanism enables slow formation of some fibrils even when both binding sites are fully occupied in most TTR molecules. Effective targeting of all TTR molecules with stabilizer drugs is much more challenging *in vivo* than *in vitro* and it is therefore crucial to consider the significant pathogenic impact of even minimal amyloid fibril formation *in vivo*. There are potentially major implications, that should not be ignored, both for interpretation of current clinical studies in TTR amyloidosis and for the design of future therapeutic approaches such as the exploitation of non-natural peptides inhibitors of TTR aggregation^16^ or antibodies recognizing cryptic epitope exposed only on the surface of aggregated TTR and not in the native tetrameric state^17^.

## Methods

### Expression of recombinant TTR

V122I TTR was expressed using a pET3a vector containing the full-length cDNA for human V122I TTR in *E. coli* BL21 super competent cells. Expression colonies were grown to an optical density at 600 nm of 1.0 in Luria-Bertani broth containing 100 µg ml^−1^ ampicillin at 37 °C; protein synthesis was induced with 1 mM isopropyl-β-D-thiogalactoside at 18 °C overnight. The following day cells were harvested by centrifugation at 2,150 *g* for 30 min, the pellet was suspended in buffer containing 25 mM Tris-HCl, 2 mM EDTA, 0.1% Triton, pH 7.4 and sonicated at 12 μm amplitude for 10 cycles (1 min on/1 min off). The intracellular proteins were fractionated by 2 cycles of ammonium sulphate precipitation. TTR, which precipitated between 30 and 60% ammonium sulphate, was dissolved in 25 mM Tris-HCl, 0.1 M NaCl, pH 8.0 and fractionated on a Superdex 75 Hi Load 26/60 gel filtration column (GE Healthcare Life Science) equilibrated and eluted in the same buffer. TTR enriched fractions were dialyzed overnight against 25 mM Tris-HCl, pH 8.0, reduced with dithiothreitol and then applied to a Q-Sepharose anion exchange column equilibrated in 25 mM Tris-HCl pH 8.0 and eluted with a linear 0–1 M NaCl gradient in the same solvent. TTR enriched fractions were pooled, concentrated and further purified on the Superdex 75 Hi Load 26/60 column. Fractions containing TTR were dialyzed against water at 4 C for at least 3 days and then lyophilized. Purity was confirmed by SDS-PAGE and electrospray ionisation mass spectrometry.

### Fibrillogenesis of V122I TTR in the presence of ligands

Fibrillogenesis experiments were performed in standard glass vials stirred at 1,500 r.p.m. (IKA magnetic stirrer) at 37 °C using 1 mg ml^−1^ of V122I TTR (18 µM tetramer) in PBS at pH 7.4 in the presence and in the absence (control) of 5 ng µl^−1^ of trypsin. Tolcapone, tafamidis and mds84 were dissolved individually at 10 mM in DMSO, followed by serial dilutions which, when added in appropriate volume to TTR, provided ligand:TTR tetramer molar ratios of 0.5:1, 1:1, 2:1 and 4:1 respectively. Turbidity at 400 nm was used to monitor fibril formation over time until it reached a plateau at 96 h. The thioflavin T assay^18^ could not be used because tolcapone and mds84 interfere with the fluorescence measurement. At the end of the aggregation, susceptibility to trypsin was monitored using SDS-homogeneous 15% PAGE (GE Healthcare) under reducing conditions. Two-way Anova was performed using GraphPad Prism 5 for pairwise multiple comparison among tafamidis, tolcapone and mds84.

The pellet was harvested from each protein sample in the absence and in the presence of ligands by ultracentrifugation in a Beckman Optima TL ultracentrifuge at 135,000 *g*, 20 min. After resuspension of the pellet with a minimal volume of PBS, samples were stained with alkaline alcoholic Congo red and examined by high intensity cross polarized light microscopy^19^. Amyloid load was scored in the Congo red stained pellet of TTR in the presence of fourfold molar excess of each ligand (Supplementary Fig. S6). A blind quantification was carried out by an expert operator on six slides per each group of treatment using the following grading score: 0 (no spot detected), 1 (occasional spots), 2 (green birefringent spots clearly visible and corresponding to the stained material in the bright field), 3 (surface homogeneously covered by green birefringent material). The non-parametric Kruskall-Wallis test for mds84 vs tafamidis (or tolcapone) was applied using GraphPad Prism 5.

Samples were also examined by negative staining transmission electron microscopy. Briefly, a drop of each sample was allowed to dry on formvar coated copper EM grids for 2 min before blotting with filter paper to remove excess solvent and staining with 2% w/v uranyl acetate for 1 min. After further blotting and drying in air, transmission electron microscope (Jeol1200EX) images were obtained at 80 kV.

### Structural analysis

X-ray structures of wild type TTR alone (PDP codes 1DVQ and 5CN3) and complexed with tolcapone, tafamidis, mds84, compound 20 and 22 (PDB codes 4D7B, 3TCT, 3IPE, 2FBR and 2FLM, respectively) were examined using VMD, SPDBV^20, 21^ and Pymol (PyMOL Molecular Graphics System, Version 1.8 Schrödinger, LLC). The energetic contribution of the linker to the binding was estimated by the SeeSAR programme (https://www.biosolveit.de/SeeSAR). The halogen binding pockets considered in the analysis are formed by Met13, Lys15, Leu17, Thr106, Ala108, Val121 (HBP1); Lys17, Ala108, Ala109, Leu110 (HBP2); Ala108, Ser117, Leu110, Thr119 (HBP3).

## Electronic supplementary material

Supplementary Information
