# Supplementary material for: Inhibition of the mechano-enzymatic amyloidogenesis of transthyretin: role of ligand affinity, binding cooperativity and occupancy of the inner channel
Source: Sci Rep. 2017 Mar 15;7:182. doi: 10.1038/s41598-017-00338-x (PMC5428290; doi:10.1038/s41598-017-00338-x)
Supplement: Supplementary file 1 — Supplementary Information [file 41598_2017_338_MOESM1_ESM.pdf]

Supplementary Information

Inhibition of the mechano-enzymatic amyloidogenesis of transthyretin: role of ligand affinity, binding cooperativity and occupancy of the inner channel

Guglielmo Verona[1], P. Patrizia Mangione[1,2], Sara Raimondi[2], Sofia Giorgetti[2], Giulia Faravelli[2], Riccardo Porcari[1], Alessandra Corazza[1,3], Julian D. Gillmore[4], Philip N. Hawkins[4], Mark B. Pepys[1,4], Graham W. Taylor[1] & Vittorio Bellotti[1,2,*]

[1]Wolfson Drug Discovery Unit, Centre for Amyloidosis and Acute Phase Proteins, University College London, London NW3 2PF, UK. [2]Department of Molecular Medicine, Institute of Biochemistry, University of Pavia, Via Taramelli 3b, Pavia 27100, Italy. [3]Department of Medical and Biological Sciences (DSMB), University of Udine , Piazzale Kolbe 4, 33100 Udine, Italy [4]National Amyloidosis Centre, University College London, London NW3 2PF, UK.

*Correspondence and requests for materials should be addressed to V.B. (email: v.bellotti@ucl.ac.uk).

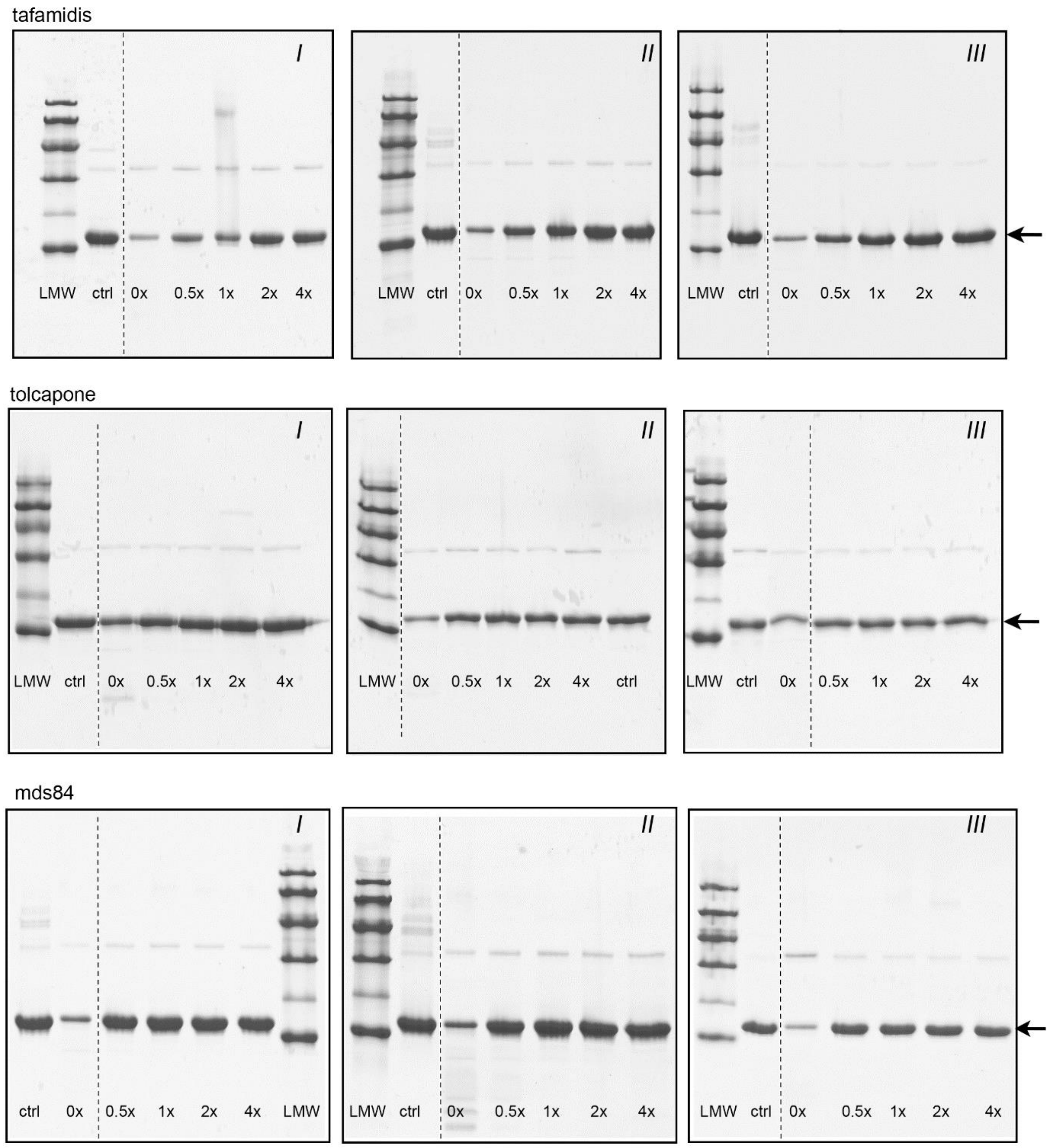

Supplementary Fig. S1. **Proteolysis of V122I TTR in the presence of TTR stabilizing drugs**. SDS-15% PAGE under reducing conditions of aliquots of 18 µM V122I TTR after 96 h aggregation in the presence of 0 (0x), 9 (0.5x), 18 (1x), 36 (2x) and 72 µM (4x) of tafamidis, tolcapone and mds84 respectively in PBS pH 7.4 at 37°C. Aggregation was carried out under fluid agitation and addition of trypsin at an enzyme: substrate ratio of 1:200.

Three replicates (*I*, *II*, *II*) for each ligand are shown. Bands corresponding to the intact TTR protomer (see arrows) were quantified with Quantity one software (Biorad). Bands corresponding to the protein before addition of trypsin (ctrl) were considered as 100% (Fig. 1b). Dashed lines indicate that lanes non adjacent in the same gel have been juxtaposed. Marker proteins (14.4, 20.1, 30.0, 45.0, 66.0 and 97.0 kDa) are included.

Inhibition of low pH TTR aggregation. V122I TTR (495 µl of 7.2 µM tetramer) in PBS, pH 7.4, and 0.1% w/v NaN3 were pre-incubated (37°C for 30 min) with 5 µl of each ligand dissolved in DMSO at increasing concentrations in order to provide a final ligand:TTR tetramer molar ratios of 0.5:1, 1:1, 2:1 and 4:1 respectively. TTR was incubated with 5 µl of DMSO as control. After the 30 min incubation period, the pH was adjusted to 4.4 by addition of 500 µl of 0.2 M sodium acetate buffer, pH 4.2. Samples were then left unstirred 72 h at 37 °C. Absorbance spectra were recorded before and after 72 h incubation (V-650 spectrophotometer; Jasco). After corresponding blank subtraction, aggregation was expressed as percent of 340-nm turbidity compared with TTR alone (100 % aggregation). All samples were performed in triplicate. Two-way ANOVA was performed using GraphPad Prism 5 to compare both tolcapone and tafamidis vs mds84.

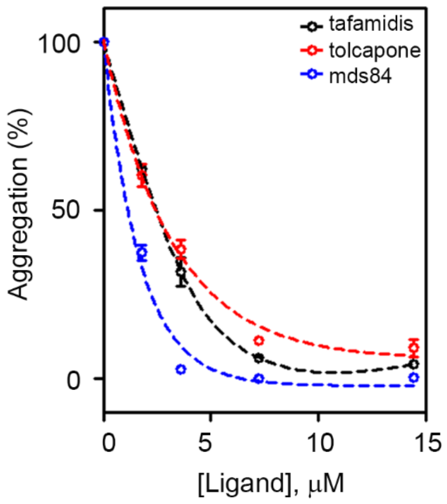

Supplementary Fig. S2. Inhibition of acidic-mediated TTR aggregation. Inhibition by tafamidis, tolcapone and mds84 of TTR aggregation at low pH. Inhibition of aggregation of V122I TTR at pH 4.4 at ligand:TTR tetramer molar ratios of 0.5:1, 1:1, 2:1 and 4:1 respectively. Data shown represent mean (SD) of three independent experiments. The

bivalent ligand mds84 is a better inhibitor than tolcapone and tafamidis ($P < 0.001$ for each concentration except for fourfold molar excess of tafamidis).

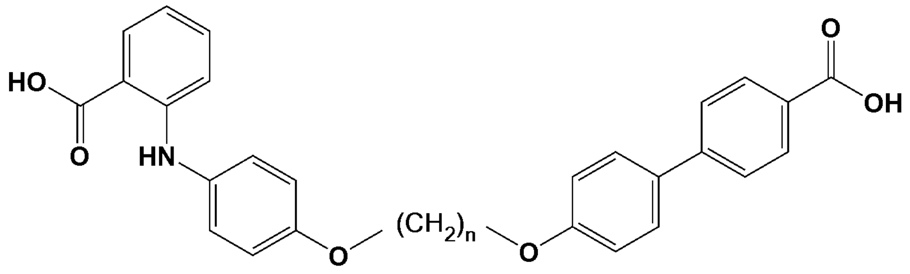

Supplementary Fig. S3. Heterobivalent compounds. Chemical structure of compounds 20 (n=4) and 22 (n=6) by Green and collaborators[1].

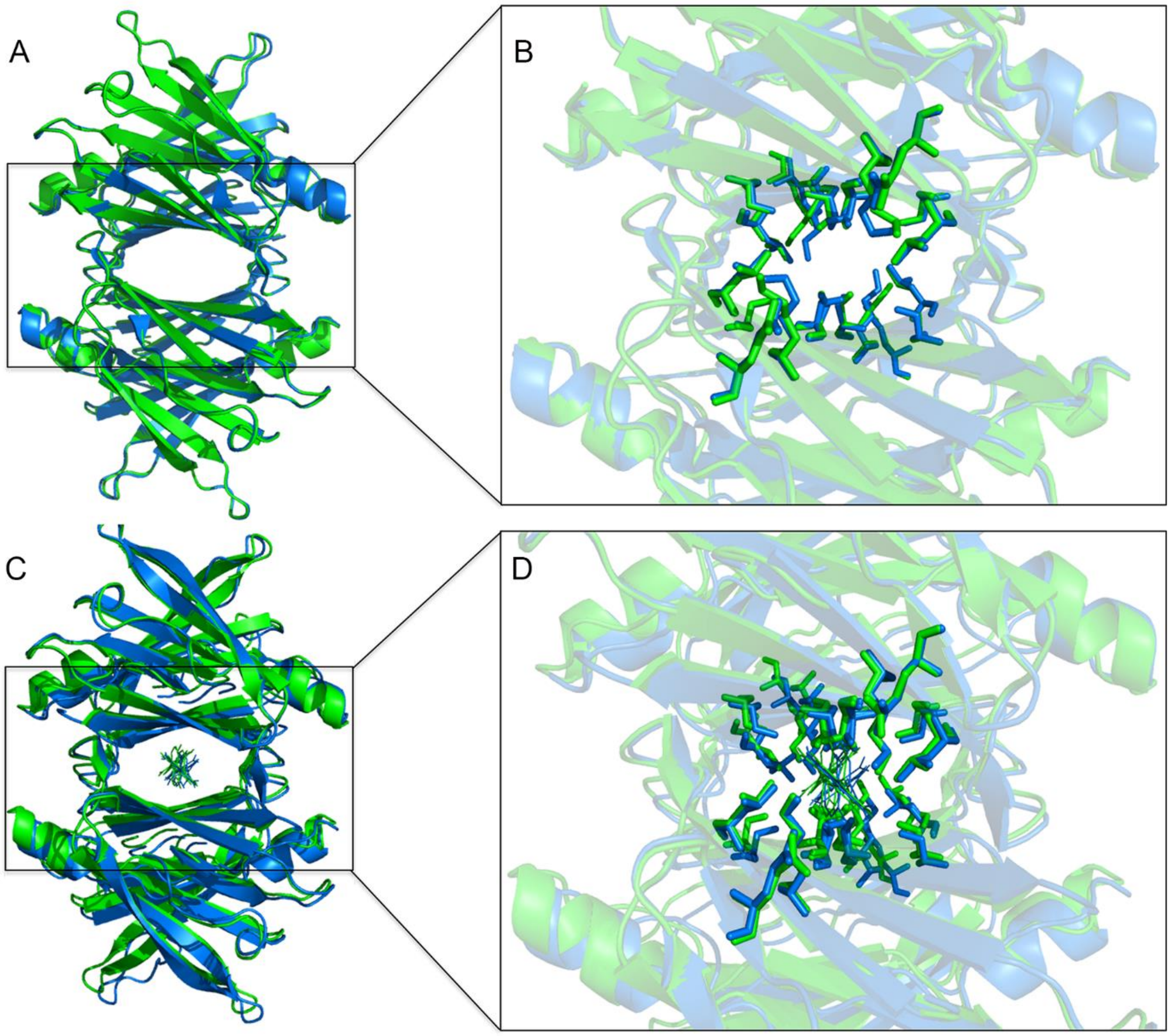

Supplementary Fig. S4. Structural comparison of wild type and V122I TTR.

Superposition of wild type, in blue, and V122I TTR, in green, in the absence (A, B) and in the presence of tolcapone (C, D). A zoomed view of the molecules with side chains of residues involved in the tolcapone binding shown as sticks in B and D. PDB codes: 5cn3, 4d7b for wild type and 1ttr and 5a6i for V122I TTR without and with tolcapone, respectively.

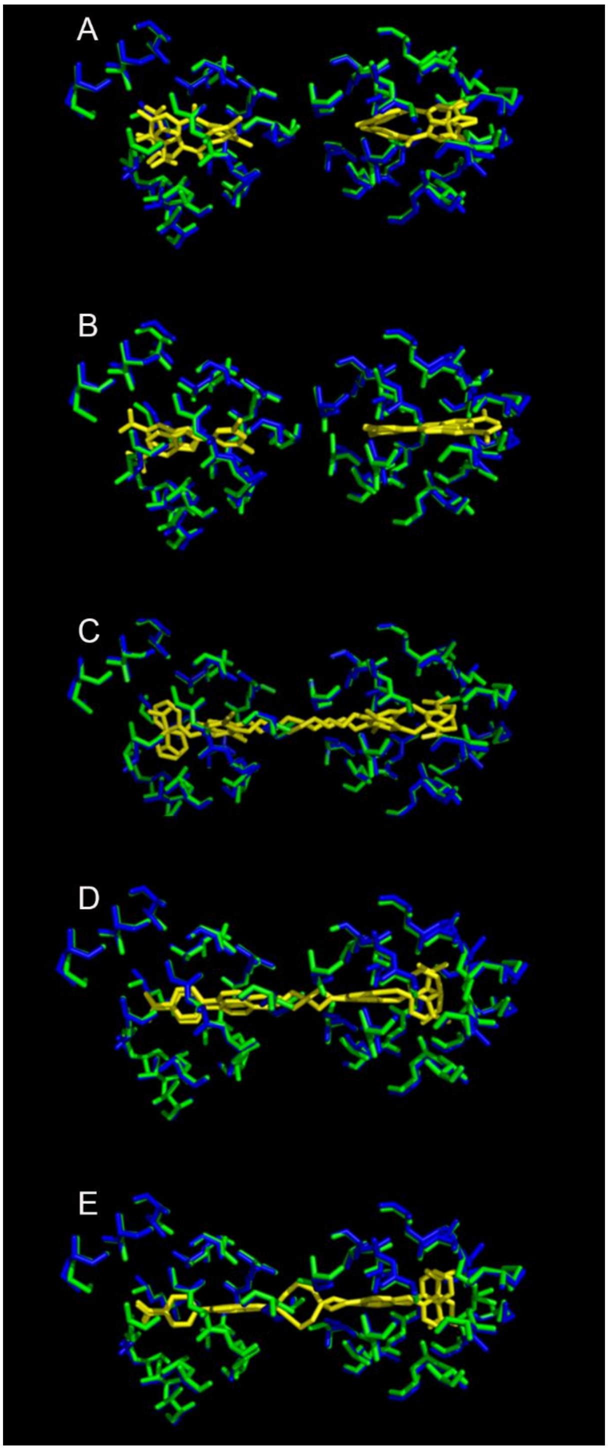

Supplementary Fig. S5. Binding pockets in the presence and absence of ligands: a structural comparison. Superposition of wild type TTR in the absence of ligands (in blue, PDB 1dvq) and in the presence of ligands in green: tolcapone (A), tafamidis (B), mds84 (C),

compound 20 (D) and compound 22 (E). Only the residues belonging to the halogen binding pockets are shown. The ligands are shown in yellow.

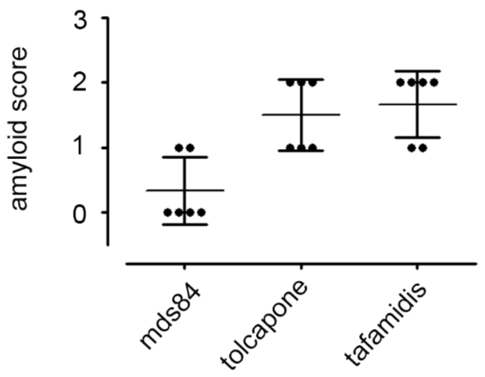

Supplementary Fig. S6. Amyloid in the ultracentrifuged TTR aggregated material. TTR was aggregated in the presence of trypsin and fourfold molar excess of ligands and was ultracentrifuged. The pellet was stained with Congo- red and analysed under light microscopy[2] for amyloid grading score. A blind quantification was carried out by an expert operator on six slides per each group of treatment using the following grading score: 0 (no spot detected), 1 (occasional spots), 2 (green birefringent spots clearly visible and corresponding to the stained spots in the bright field), 3 (surface homogeneously covered by green birefringent material). Kruskall-Wallis test for mds84 vs tafamidis (or tolcapone) was applied showing statistical significance ($P < 0.05$) for each corresponding pair.

Supplemetary Table S1. List of hydrogen bonds between ligands and the TTR binding pocket.

Ligand atom*	TTR atom**	H bond distance (Å)
**Wild type TTR + tolcapone (PDB: 4d7b)**		
A/TCW 1126/O11	D/LYS`/15/NZ	3.12
B/TCW 1126/O10	C/LYS`/15/NZ	3.10
C/TCW 1126/O11	B/LYS`/15/NZ	3.12
**Wild type TTR+ tafamidis (PDB: 3tct)**		
no hydrogen bonds		
**Wild type TTR + mds84 (PDB:3ipe)**		
B/JZE 128/O3	C/SER /117/OG	3.03
D/JZE 128/O3	A/SER /117/OG	3.03
**Wild type TTR+ Compound 20 (PDB: 2fbr)**		
A/44C 173/O14	D/LYS /15/NZ	2.41
A/44C 173/O15	B/LYS /15/NZ	2.46
A/44C 173/O21	C/SER/ 117/OG	2.57
C/44C 173/O14	B/LYS /15/NZ	2.41
C/44C 173/O15	D/LYS/ 15/NZ	2.46
C/44C 173/O21	A/SER /117/OG	2.57
**Wild type TTR + Compound 22 (PDB: 2flm)**		
no hydrogen bonds		

* chain/ligand/ atom name

** chain/residue /residue number/atom name

Supplementary references

1 Green, N. S., Palaninathan, S. K., Sacchettini, J. C. & Kelly, J. W. Synthesis and characterization of potent bivalent amyloidosis inhibitors that bind prior to transthyretin tetramerization. *J. Am. Chem. Soc.* **125**, 13404-13414, (2003).

2 Puchtler, H., Waldrop, F. S. & Meloan, S. N. A review of light, polarization and fluorescence microscopic methods for amyloid. *Appl. Pathol.* **3**, 5-17 (1985).
